# Loss of genetic diversity as a consequence of selection in response to high *p*CO_2_

**DOI:** 10.1111/eva.12404

**Published:** 2016-07-27

**Authors:** Melanie M. Lloyd, April D. Makukhov, Melissa H. Pespeni

**Affiliations:** ^1^ Department of BiologyUniversity of VermontBurlingtonVTUSA

**Keywords:** alternative splicing, nucleotide diversity, ocean acidification, RNA sequencing

## Abstract

Standing genetic variation may allow for rapid evolutionary response to the geologically unprecedented changes in global conditions. However, there is little known about the consequences of such rapid evolutionary change. Here, we measure genetic responses to experimental low and high *p*CO_2_ levels in purple sea urchin larvae, *Strongylocentrotus purpuratus*. We found greater loss of nucleotide diversity in high *p*CO_2_ levels (18.61%; 900 μatm) compared to low *p*CO_2_ levels (10.12%; 400 μatm). In the wild, this loss could limit the evolutionary capacity of future generations. In contrast, we found minimal evidence that purple sea urchin larvae physiologically respond to high *p*CO_2_ through alternative splicing of transcripts (11 genes), despite a strong signal of alternative splicing between different developmental stages (1193 genes). However, in response to high *p*CO_2_, four of the 11 alternatively spliced transcripts encoded ribosomal proteins, suggesting the regulation of translation as a potential response mechanism. The results of this study indicate that while the purple urchin presently may have enough standing genetic variation in response to rapid environmental change, this reservoir of resilience is a finite resource and could quickly diminish.

## Introduction

Increasing atmospheric carbon due to anthropogenic activities lowers ocean pH causing changes to ocean carbonate chemistry (Doney et al. 2009; Hönisch et al. 2012). The consequences of ocean acidification for many ecologically and economically important marine organisms are slower growth, reduced fecundity, and higher mortality (Guinotte and Fabry 2008; Kroeker et al. 2010; Gaylord et al. 2011). To avoid such negative impacts of fast‐changing environmental conditions, organisms will have to move to more favorable conditions, physiologically acclimatize, or genetically adapt (Somero 2010; Hoffmann and Sgrò 2011).

The ability of organisms with long generation times to rapidly adapt depends upon the amount of variation in present populations (Lande and Shannon 1996; Barrett and Schluter 2008). Past evolution in a temporally or spatially heterogeneous environment could allow for the maintenance of adequate standing genetic variation and physiological plasticity in present‐day populations required for surviving in projected future acidification conditions (Levene 1953; Lande and Shannon 1996; Dupont et al. 2010; Sunday et al. 2014). Many species along the west coast of North America, including the purple sea urchin, *Strongylocentrotus purpuratus*, have evolved in a mosaic of seasonally and spatially variable temperature and pH due to the natural process of wind‐driven upwelling of cold, low‐pH waters from the deep ocean (Menge 2000; Feely et al. 2008). In a previous study, we found minimal negative morphological effects of high *p*CO_2_ enabled by selective survival of larvae with favorable alleles in high *p*CO_2_ conditions in genes related to ion homeostasis, lipid metabolism, and biomineralization (Pespeni et al. 2013b), supporting the hypothesis that evolution in a variable *p*CO_2_ environment could allow for adequate genetic variation to respond to increased *p*CO_2_. A common consequence of rapid adaptation is decreased genetic diversity in surviving populations, which would decrease future generations’ adaptive potential (Rodríguez‐Trelles and Rodríguez 1998; Jump and Penuelas 2005; Jump et al. 2009). In this study, we tested the hypothesis that the changes in allele frequency observed in the purple sea urchin in response to increased *p*CO_2_ resulted in decreased genetic diversity.

Results from this previous analysis indicate purple urchin larvae show minimal gene expression responses to elevated *p*CO_2_: only 32 genes were differentially up‐ or downregulated in experimentally acidified conditions (Pespeni et al. 2013b). This minimal response could have several potential explanations. Because the elevated *p*CO_2_ treatment (900 μatm) was within the range of what is experienced on occasion in nature with upwelling (Feely et al. 2008; Hofmann et al. 2011), it could be that the stressor, while strong enough to elicit selective survival of larvae with specific allelic variants, does not warrant a strong physiological gene expression response. Alternatively, it could be that the physiological responses involved other mechanisms that might not be detected through differences in transcript abundance, such as solute regulation, enzyme activities, post‐translational modifications, or alternative splicing of transcripts (Pörtner and Farrell 2008; Somero 2012; Pan et al. 2015). Alternative splice variation allows for structurally and functionally different proteins to be produced from a single gene or locus, providing functional diversity that exceeds what is possible through differential regulation of transcript abundance (Marden 2008; Irimia et al. 2009). In addition, differences in expression of alternative splice variants are not detected in standard gene expression analyses. To broaden our understanding of the tools available to purple urchin larvae to respond to acidified conditions, we tested their ability to physiologically acclimate through alternative splice variation.

Purple urchins have evolved in a heterogeneous environment that may have facilitated the accumulation of adequate genetic variation for selective survival in high *p*CO_2_ conditions and/or the physiological capacity to acclimate to high *p*CO_2_. In this study, we tested the hypothesis that the selective mortality in high *p*CO_2_ conditions would result in decreased genetic diversity in the surviving population. We further investigated the ability of the purple sea urchin to physiologically acclimate to high *p*CO_2_ through alternative splice variation rather than differential gene regulation.

## Methods

### Collection of sequence data

RNA‐sequencing data were generated in a previous acidification experiment (Pespeni et al. 2013b). In the previous study, adult sea urchins were collected from seven populations along the species range (Northern California: Bodega Bay and Van Damme; Oregon: Strawberry Hill and Fogarty Creek; Central California: Sand Hill Bluff, Terrance Point; and Southern California: Alegria). For each population, eggs from each of 10 females were fertilized by sperm from 10 males. Resulting embryos were reared in either low (400 μatm, pH 8.01 ± 0.03) or high (900 μatm, pH 7.72 ± 0.03) *p*CO_2_ conditions. These high *p*CO_2_ conditions are experienced in nature along the purple sea urchin species range due to the natural process of upwelling of low‐pH waters from the deep ocean (Feely et al. 2008; Hofmann et al. 2011). Larvae were sampled every 2 days through metamorphosis (~50 days) for morphometric analyses. For each population and each treatment, pools of ~1000 larvae were sampled from additional replicate cultures at 1 and 7 days postfertilization for RNA sequencing. RNA‐sequencing libraries were prepared for each sample (~1000 larvae per population per treatment per developmental time point) using Illumina's TruSeq kit (San Diego, CA, USA). Each library was sequenced on a single Illumina HiSeq lane yielding ~80 million quality‐filtered 50‐bp single‐end reads per sample. More details about the methods used to generate this RNA‐sequencing data and the water chemistry data can be found in the original publication(Pespeni et al. 2013b).

To identify consistent responses to *p*CO_2_ across populations, we used the sequence data from seven populations as replicates for each of the four conditions: low and high *p*CO_2_ at 1 and 7 days postfertilization: day 1–400 μatm (D1‐400), day 1–900 μatm (D1‐900), day 7–400 μatm (D7‐400), and day 7–900 μatm (D7‐900). While there were no morphological or developmental differences between populations, previous results found a correlation between the allele frequencies at 318 SNPs and local pH conditions (Pespeni et al. 2013a). It is possible that cellular or metabolic differences may not have been captured by morphological and developmental metrics (Pan et al. 2015). As a consequence, there may have been population‐specific responses that were not captured in our analysis.

### Nucleotide diversity

Starting with quality‐filtered RNA‐sequencing reads, we mapped reads to the *S. purpuratus* genome version 3.1 (Sodergren et al. 2006) using TopHat2 default parameters (max read mismatches 2, max gap length 2, max insertion/deletion 3, and min anchor length 8) (Trapnell et al. 2009; Kim et al. 2013). The resulting bam files produced one vcf file with VCFtools (Danecek et al. 2011). We filtered the vcf file (minimum depth: 50, maximum missing data: 0.8, minimum/maximum alleles: 2, and minGQ:20 corresponding to a 99% confidence of correctly identified genotype, indels removed) which was used to identify the location of all SNPs. We produced a separate vcf file per treatment group (Day1‐400, D1‐900, D7‐400, and D7‐900) and estimated nucleotide diversity (measured as *π*) on windows of 10 000 base pairs. We tested the difference in nucleotide diversity due to the effect of day, *p*CO_2_ level, and the interaction between day × *p*CO_2_ level using permutation analysis. First, metrics of *π* were log‐transformed to meet assumptions of normality. The values of log(*π*) of each window were randomly shuffled 10 000 times across all windows of the entire dataset and reassigned to treatment group. For each of the 10 000 permutations, we tested for the effect of day, *p*CO_2_ level, and the interaction between day × *p*CO_2_ level using an anova. We compared the permuted *P*‐values to the observed *P*‐value for each factor. Significance was determined as the proportion of permuted calculations less than the observed. The custom R script is provided in Data S1.

### Accounting for allelic bias

Allelic mapping bias, where reads containing reference alleles at polymorphic sites will map more efficiently than reads containing alternative alleles at the same site, can confound differential expression analyses from short read RNA‐sequencing datasets (Degner et al. 2009; van de Geijn et al. 2015). We used WASP to identify and remove any reads containing reference alleles which specifically contributed to allelic mapping bias (van de Geijn et al. 2015). We used the bam files output from Tophat2 to produce one vcf file with VCFtools (Danecek et al. 2011) which was filtered and used to identify the location of all SNPs as described above. We used WASP to identify and remove reads which contained reference alleles which otherwise would not have mapped had they contained alternative alleles. We re‐assembled all remaining reads with TopHat2 using the same default parameters.

It is worth noting that WASP only removes data that would contribute to allelic mapping bias by removing reads; it does not improve mapping by correctly mapping reads containing alternative alleles. Uncorrected allelic mapping bias would only incorrectly underestimate nucleotide diversity estimates because alternative alleles would map less frequently than they truly occur. Furthermore, we were interested in proportional differences in nucleotide diversity in *p*CO_2_ treatments through developmental time. Inefficient mapping of alternative alleles compared to reference alleles would be equal in all four treatment groups. False‐negative detection of polymorphisms would be equally probable in all four treatment groups. Therefore, polymorphic sites in day 7 treatments would not be disproportionally undetected. Thus, we used the quality‐filtered, rather than WASP‐filtered, data to estimate nucleotide diversity and the WASP‐filtered data in the following analysis of differential expression of alternative splice variants.

### Differential expression of alternative splice variants

In the previous analysis of this dataset, only 32 genes were found to be differentially expressed due to *p*CO_2_ treatment. In this study, we investigated the possibility of differences in transcription arising from differentially expressed alternative splice variants that would be missed in standard differential gene expression analysis, which counts the number of reads that map to single transcripts representing gene sequences. We used DEXSeq to test for differential expression of alternative splice variants between treatment groups (Anders et al. 2012). DEXSeq tests for differential usage of individual exons in relation to the other exons in the same gene model. This eliminates the necessity of assembling entire transcripts across potentially complicated gene models. We used Python scripts provided with DEXSeq to align WASP‐filtered reads to defined exon bins of each gene model from the *S. purpuratus* transcriptome (Cameron et al. 2009; Tu et al. 2012, 2014). Expression data (counts per exon) were normalized. We tested the following predictors of differential exon usage individually: the effect of day, the effect of *p*CO_2_ level, the effect of *p*CO_2_ level accounting for the effect of day, and the interaction between day × *p*CO_2_ level. Full and reduced models for each predictor were compared as follows – effect of day: reduced model ~ sample + exon, full model ~ sample + exon + day × exon; effect of *p*CO_2_ level: reduced model ~ sample + exon, full model ~ sample + exon + *p*CO_2_ level × exon; effect of *p*CO_2_ level accounting for day: reduced model ~ sample + exon + day × exon, full model ~ sample + exon + day × exon + *p*CO_2_ level × exon; effect of the interaction between day and *p*CO_2_ level: reduced model ~ sample + exon + day × exon + *p*CO_2_ level × exon, full model ~ sample + exon + day × exon + *p*CO_2_ level × exon + day × *p*CO_2_ level × exon. We produced expression plots for each gene with significant differential exon usage (*P *<* *0.1). To test whether any gene categories were significantly enriched in this analysis, we performed a rank‐based gene ontology analysis with adaptive clustering using the negative log transformation of the Benjamini Hochberg adjusted *P* value from each of the four above tested models (Dixon et al. 2015). Unlike other gene ontology enrichment analyses, this does not test for enrichment within a list of significant genes, but rather uses the whole distribution in a rank‐based test to identify GO terms enriched along a continuous metric such as *P*‐value. All code used is provided in Data S1.

## Results

### Nucleotide diversity

**Figure 1:**
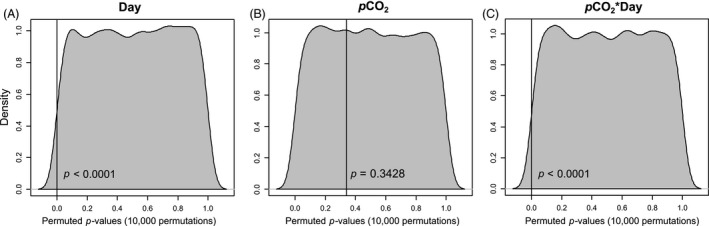
Permutation density plots of an anova to test the significance of (A) day, (B) *p*CO_2_ level, and (C) the interaction between day × *p*CO_2_ level on nucleotide diversity as measured by *π*. Horizontal lines mark the observed *P*‐value for each factor.

The overall nucleotide diversity (*π*) of all data was 0.00022 from 503 564 SNPs. There was a significant decrease in nucleotide diversity between day 1 and day 7 in both the low and high *p*CO_2_ treatment groups; however, there was a higher proportion of diversity lost in the high *p*CO_2_ group (18.61%) compared to the low *p*CO_2_ group (10.12%, Table 1). Permutation analyses indicate that the effect of day (*P *<* *0.0001) and the interaction between day × *p*CO_2_ level (*P *<* *0.0001) are both highly significant factors of nucleotide diversity (Fig. 1). Decreased genetic diversity between day 1 and day 7 was also measured as the number of polymorphic SNPs in each of the four treatment groups. Similar to the loss of diversity, as measured by *π*, the number of polymorphic SNPs decreased between day 1 and day 7 in both the low and high *p*CO_2_ treatment groups. Again, there was a larger proportion of SNPs lost in the high *p*CO_2_ group (8.15%) compared to the low *p*CO_2_ group (5.87%, Table 1).

**Table 1:** Summary statistics of nucleotide diversity (*π*) and number of SNPs

	Low *p*CO_2_	High *p*CO_2_
Day 1		
Mean *π*	0.00107	0.00114
Number of SNPs	480 932	497 167
Day 7		
Mean *π*	0.00097	0.00093
Number of SNPs	452 687	456 671
Percent lost through time		
Mean *π*	10.12	18.61
Number of SNPs	5.87	8.15

### Differential expression of alternative splice variants

**Figure 2:**
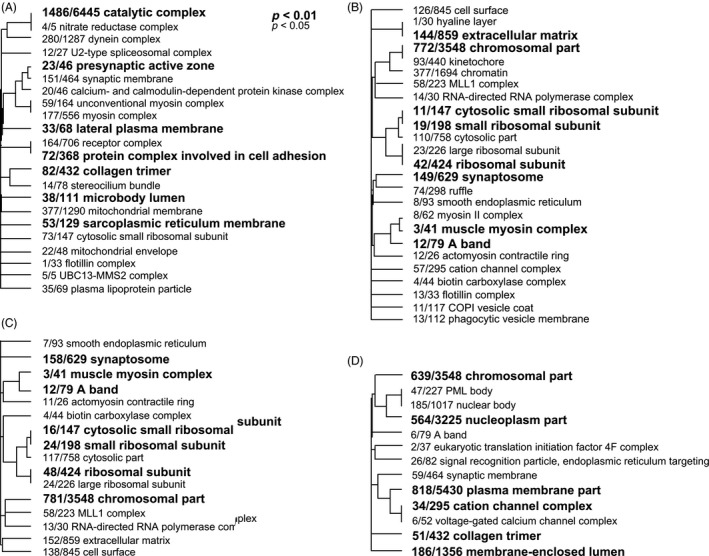
Enriched GO terms of genes with differential exon usage between (A) day, (B) *p*CO_2_ levels, (C), *p*CO_2_ levels accounting for the difference between days, and (D) the interaction between day × *p*CO_2_ level. Dendrograms indicate shared genes among GO terms; fractions indicate the number of genes with uncorrected *P *<* *0.05 compared to the total number of genes in a category. Only enriched GO terms with FDR *P *<* *0.05 were included on the figure.

We identified 119 786 exons from 21 108 genes in our RNA‐sequencing dataset. In total, 2076 exons belonging to 1201 genes were differentially expressed in our analyses. DEXSeq identifies differentially expressed exons in relation to other exons within the same gene model. This accounts for the overall expression differences at the gene level. Differential expression of one or more exon within one gene between treatments implies alternative splice variation within that gene (see Figure S1 for an example output of differential exon usage, gene ID WHL22.665129: SPU_010393, telomere elongation helicase‐like). The vast majority of genes with differential exon usage (1193 genes) were alternatively spliced between day 1 and day 7 and represent the alternative splice variation between two distinct developmental stages in purple urchin larvae, hatched blastula, and four‐arm plutei (Table S1). Among the genes with differentially expressed exons, the average number of differentially expressed exons per gene was 1.73, indicating that alternatively spliced transcripts may result from only one or two exon changes. There were far fewer differentially expressed exons in different *p*CO_2_ treatments: only nine exons belonging to eight genes were differentially expressed due to *p*CO_2_ level alone (all low *p*CO_2_ versus all high *p*CO_2_ samples); four exons belonging to three genes were differentially expressed due to *p*CO_2_ level while accounting for the effect of day; and one exon belonging to one gene was differentially expressed due to the interaction between day × *p*CO_2_ level (Tables 2 and 3). We also used enrichment analyses to identify suites of genes related by biological function that were alternatively spliced in these models. We found a further 41 (day), 37 (condition), 36 (condition accounting for day), and 42 (condition × day) GO terms enriched in these respective analyses (FDR < 0.1). Genes with significant differential exon usage between *p*CO_2_ level were largely ribosomal genes (Table 3). Furthermore, while the number of enriched GO terms was similar in the model testing for the effect of day and the three models testing for the effects of *p*CO_2_ level, there was a clear pattern where ribosomal and translation genes were enriched in models testing for the effects of *p*CO_2_ level, but not enriched in the model testing for the effect of day (Fig. 2).

**Table 2:** Summary statistics of differential exon expression (FDR *P *<* *0.05)

Effect tested	Exons differentially expressed	Genes with differentially expressed exons	Number of GO terms enriched (FDR *P *<* *0.1)
Day	2067	1193	41
*p*CO_2_ level	9	8	37
*p*CO_2_ level accounting for day	4	3	36
*p*CO_2_ level × day	1	1	42

**Table 3:** Annotations of genes with significant differential exon expression specific to *p*CO_2_ level

WHL code	Significant Factors	Annotation
22.38329	*p*CO_2_ level	Nucleoporin 205 kDa and membrane progestin receptor gamma
22.442792	*p*CO_2_ level	Ribosomal protein S2
22.445772	*p*CO_2_ level	Ribosomal protein S7
22.665129	*p*CO_2_ level	Telomere elongation helicase‐like
22.510905	*p*CO_2_ level, day	Apolipoprotein B
22.262267	*p*CO_2_ level, day	Folylpolyglutamate synthase
22.667356	*p*CO_2_ level, day	Ribosomal protein L5
22.648077	*p*CO_2_ level, *p*CO_2_ level accounting for day	Tumor protein, translationally controlled 1
22.241670	*p*CO_2_ level accounting for day	ATP synthase, H+ transporting, mitochondrial F1 complex, alpha subunit
22.186040	*p*CO_2_ level accounting for day	Ribosomal protein L23a
22.563676	*p*CO_2_ level × day	Aristaless‐like homeobox 1‐like, Cart1/Alx3/Alx4 subfamily‐like

## Discussion

The results of this study show decreased nucleotide diversity following evolutionary change in response to increased *p*CO_2_ in purple sea urchin larvae. There was significant loss in genetic diversity of cultured larvae between day 1 and day 7 in both high and low *p*CO_2_ conditions; however, the percent nucleotide diversity lost in high *p*CO_2_ was significantly more than in low *p*CO_2_ (*P* < 0.0001, Table 1). *p*CO_2_ treatment alone, pooled across days, was not a significant predicting factor of nucleotide diversity, indicating the negative effect of increased *p*CO_2_ acts through developmental time (Fig. 1). A similar pattern was seen in the total number of polymorphic SNPs of the four experimental treatment groups. The total number of polymorphic SNPs decreased between day 1 and day 7 in both high and low *p*CO_2_ conditions; however, the number of polymorphic SNPs lost in high *p*CO_2_ was significantly more than in low *p*CO_2_. SNPs that go to fixation will lower standing genetic diversity in a population and be lost except in the event of dispersal of individuals carrying unique genetic variants or until a new neutral or beneficial mutation occurs.

Previous experimental results from which our dataset arose concluded that the larvae reared in high *p*CO_2_ did not show any major differences in morphology and growth rates compared to larvae in low *p*CO_2_. Overall, larvae at high *p*CO_2_ were 4–5% smaller in length, but larvae in both conditions were equally able to metamorphose, indicating no substantial difference in developmental trajectory (Pespeni et al. 2013b). Our previous study also identified changes in allele frequencies and amino acid changing polymorphisms through developmental time in specific classes of genes related to ion homeostasis, lipid metabolism, and biomineralization in larvae reared in high *p*CO_2_ while changes were random with respect to protein function in low *p*CO_2_. These results suggest there is adequate present‐day genetic diversity to allow for selective survival of larvae with specific alleles well suited for high *p*CO_2_. To add to this conceptual model, in this study, we found that the nucleotide diversity of the surviving larvae had decreased more in high *p*CO_2_ than for larvae reared in low *p*CO_2_, 83% more loss of diversity in high *p*CO_2_ compared to low (18.61% vs 10.12%)_._ We now have a more complete understanding of the evolutionary effects of high *p*CO_2_ on the purple sea urchin: rapid evolutionary change leading to decreased genetic diversity with few transcriptional changes.

### Consequences of reduced nucleotide diversity

Generally, the present‐day populations of purple urchins have high genetic diversity (Pespeni and Palumbi 2013), but the results of this study indicate that this could change in the near future due to anthropogenic environmental changes. Genetic diversity has a fundamental role in the future evolutionary trajectory of a species. Just as present‐day populations need a high genetic diversity to rapidly adapt, future generations will need equally as much genetic diversity to adapt to future changes (Barrett and Schluter 2008). Reduced genetic diversity has been shown to decrease disease resistance (Spielman et al. 2004) as well as resilience to environmental disturbance (Hughes and Stachowicz 2004) and extreme temperatures (Reusch et al. 2005). Although the genetic variation is currently high, the rapid rate of evolutionary change could outpace the rate of adaptation of this species. Future studies are necessary to understand how the loss of genetic diversity will impact the ability of future generations to continue to cope with environmental change in the purple sea urchin.

### Alternative splicing

In accord with our previous differential expression analysis of the dataset (Pespeni et al. 2013b), the present study found very few transcripts differentially expressed between *p*CO_2_ levels. The number of significantly alternatively spliced genes (FDR *P *<* *0.1) was dramatic between days, but not between *p*CO_2_ levels (Table 2). There was no overlap between the genes significantly differentially expressed in the previous analysis and the genes significantly alternatively spliced in this analysis. Interestingly, four of the eleven alternatively spliced genes between *p*CO_2_ conditions were ribosomal genes (Table 3, Fig. 2). This may suggest physiological changes in the purple urchin in response to *p*CO_2_ are not transcriptionally regulated, but regulated at the post‐transcription or translation levels and merits further investigation.

Recent studies show the regulation of ribosomes may be highly specialized. Alternative splicing of ribosomal mRNA may lead to specialized ribosomes which have much larger downstream effects on the translation of the entire proteome than the simple up‐ or downregulation of single gene transcripts (Xue and Barna 2012). There are many paralogous gene copies encoding ribosomal proteins, although it remains controversial to what extent the paralogs are biologically functional (Xue and Barna 2012; Zhang et al. 2013). It is possible that the difference in exon usage seen here in this study is due to transcription of multiple paralogous ribosomal genes and not alternative splice variation of single genes.

### Response to a high *p*CO_2_ level that is within the natural range

As mentioned, previous results from which our dataset arose concluded that the larvae reared in high *p*CO_2_ showed minimal morphological effects (4–5% reduction in body length) and no developmental delay compared to larvae reared in low *p*CO_2_ (Pespeni et al. 2013b). These results differ from some previous reports of negative effects of elevated *p*CO_2_ on sea urchin growth and development in acidified conditions (O'Donnell et al. 2009; Stumpp et al. 2011; Byrne et al. 2013), but not others (Yu et al. 2011). Some previous studies found stronger negative effects of increased *p*CO_2_ on the morphology and development of sea urchin larvae (O'Donnell et al. 2009; Stumpp et al. 2011; Byrne et al. 2013). However, similar to our results, Yu et al. (2011) reported that larvae reared at *p*CO_2_ 1000 μatm decreased in size by <7% (exact number not reported) compared to larvae reared in low *p*CO_2_ which is comparable to the 4–5% size decrease seen here. It is possible that these discrepancies were due to differences in densities of larval culture. Our larval cultures were 10 times less dense than those used in the studies mentioned and may be more ecologically relevant (Strathmann 1987). We hypothesize that the higher larval densities used in other studies may have interacted with elevated *p*CO_2_ to exacerbate the negative effects on developing larvae, although this remains to be tested. Effects of increased *p*CO_2_ have been shown to interact with such effects as food availability, where the overall effect of acidification is much greater in food‐limited urchin larval cultures (Pan et al. 2015).

The minimal negative effects of high *p*CO_2_ observed in this study may have been because the experimental high *p*CO_2_ condition was not high enough to elicit a transcriptional response in our experimental conditions. Purple urchins along the west coast of North America have evolved in a heterogeneous environment of seasonally and spatially variable temperature and pH due to the natural process of wind‐driven upwelling of cold, low‐pH waters from the deep ocean (Menge 2000; Feely et al. 2008). They naturally experience pH conditions similar to that of our high *p*CO_2_ experimental treatment daily or weekly (Hofmann et al. 2011; Yu et al. 2011). It is possible that they are able to manage surviving in high *p*CO_2_ conditions up to a point, higher than we used in this study (Dorey et al. 2013). In the future, it will be important to investigate these responses in higher *p*CO_2_ conditions as well as more variable *p*CO_2_ conditions. While living in a variable environment likely allows for the maintenance of high genetic variation, it may not necessarily translate into higher survivorship in the future ocean. Future global change will come with increased variability and lower predictability, pushing organisms outside of their physiological limit at some times, but not at others (Dorey et al. 2013). This unpredictable environment may be the most difficult factor to adapt to (Hamdoun and Epel 2007).

Understanding the ability as well as the long‐term effect of rapid adaptation in the face of near future environmental stressors is essential to understanding long‐term species survival. This information is necessary to identify potential ‘winners’ and ‘losers’ in an environment with higher *p*CO_2_, changing climate, and acidifying ocean. Results of our previous paper taken with this study indicate that genetic diversity is required to adapt to anthropogenic environmental changes, but is subsequently lost through this process of adaptation. The conservation of this genetic diversity within species is critical because of the expectation that its loss could render populations and species less able to adapt to ongoing or new environmental changes.

## Data archiving statement

Raw data used in this study have been archived in the NCBI Sequence Read Archive database (Acc. No. SRA SRP075627).

## Supporting information


**Figure S1.** An example output of DEXSeq for significant gene WHL 22.665129.

**Table S1.** DEXSeq results table for comparison of differential exon expression between day 1 and day 7.

**Data S1.** Includes all code used for data analysis.
