# Supplementary figures and images for: Loss of genetic diversity as a consequence of selection in response to high pCO 2
Source: Evol Appl. 2016 Jul 27;9(9):1124–32. doi: 10.1111/eva.12404 (PMC5039325; doi:10.1111/eva.12404)

# WHL22.665129 +

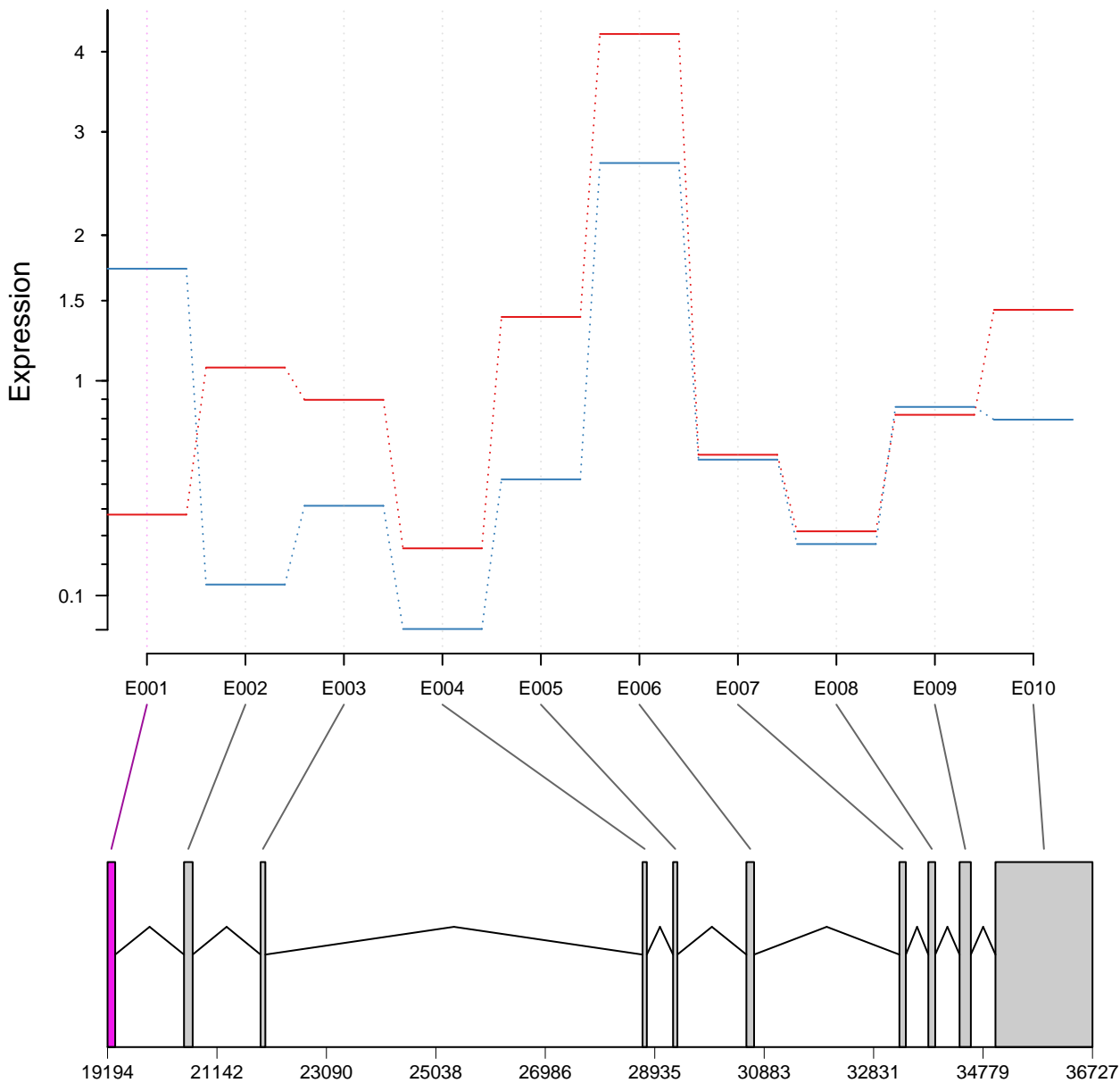

Supplement: Supplementary file 1 — Figure S1. An example output of DEXSeq for significant gene WHL 22.665129. [file EVA-9-1124-s001.pdf]
